# Impact of treatment planning target volumen (PTV) size on radiation induced diarrhoea following selenium supplementation in gynecologic radiation oncology - a subgroup analysis of a multicenter, phase III trial

**DOI:** 10.1186/1748-717X-8-72

**Published:** 2013-03-25

**Authors:** Ralph Muecke, Oliver Micke, Lutz Schomburg, Jens Buentzel, Michael Glatzel, Dieter Baaske, Regina Berndt-Skorka, Franz J Prott, Berthold Reichl, Klaus Kisters, Ulrich Schaefer, Jutta Huebner, Hans Th Eich, Guenther Kundt, Irenaeus A Adamietz

**Affiliations:** 1 Department of Radiotherapy, Lippe Hospital, Lemgo, Germany; 2 Department of Radiotherapy and Radiation Oncology, Franziskus Hospital, Bielefeld, Germany; 3 Institute for Experimental Endocrinology, Charité, Berlin, Germany; 4 Department of Otolaryngology, Südharz Hospital, Nordhausen, Germany; 5 Department of Radiotherapy, Municipal Hospital, Erfurt, Germany; 6 Department of Radiotherapy, Municipal Hospital, Chemnitz, Germany; 7 Department of Radiotherapy, Municipal Hospital, Neubrandenburg, Germany; 8 Department of Radiotherapy, St. Josefs Hospital, Wiesbaden, Germany; 9 Department of Radiotherapy, Municipal Hospital, Weiden, Germany; 10 Department of Internal Medicine, St. Anna Hospital, Herne, Germany; 11 Working Group Integrative Oncology, Dr. Senckenberg Chronomedical Institute,J.W. Goethe University, Frankfurt, Germany; 12 Department of Radiotherapy and Radiation Oncology, Universitiy of Muenster, Muenster, Germany; 13 Institute for Biostatistics and Informatics in Medicine and Ageing Research, University of Rostock, Rostock, Germany; 14 Department of Radiotherapy and Radiation Oncology, Marien Hospital Herne, Ruhr University, Bochum, Germany

**Keywords:** Planning target volume, Radiation induced diarrhoea, Selenium administration in gynecologic radiation oncology

## Abstract

**Background:**

In a previous analysis (Int J Radiat Oncol Biol Phys 70:828-835,2010), we assessed whether an adjuvant supplementation with selenium (Se) improves Se status and reduces the radiation-induced side-effects of patients treated by adjuvant radiotherapy (RT) for cervical and uterine cancer. Now, a potential relation between the planning target volume (PTV) of the RT and the Se effect concerning radiation induced diarrhoea was evaluated in detail.

**Methods:**

Whole blood Se concentrations had been measured in patients with cervical (n=11) and uterine cancer (n=70) after surgical treatment, during, and at the end of RT. Patients with initial Se concentrations of less than 84 μg/l were categorized as Se-deficient and randomized before RT to receive Se (as sodium selenite) per os on the days of RT, or to receive no supplement during RT. Diarrhoea was graded according to the Common Toxicity Criteria system (CTC, Version 2a). The evaluation of the PTV of the RT was ascertained with the help of a specialised computer-assisted treatment planning software used for radiation planning procedure.

**Results:**

A total of 81 patients had been randomized for the initial supplementation study, 39 of which received Se [selenium group, SeG] and 42 serving as controls [control group, CG]. Mean Se levels did not differ between SeG and CG upon study initiation, but were significantly higher in the SeG compared to the CG at the end of RT. The actuarial incidence of at least CTC 2 radiation induced diarrhoea in the SeG was 20.5% compared to 44.5% in the CG (p=0.04). The median PTV in both groups was 1302 ml (916–4608). With a PTV of <= 1302 ml (n=41) the actuarial incidence of at least CTC 2 diarrhoea in the SeG was 22.3% (4 of 18 patients) compared to 34.8% (8 of 23 patients) in the CG (p=0.50). In patients with a PTV of > 1302 ml (n=40) the actuarial incidence of at least CTC 2 diarrhoea in the SeG was 19.1% (4 of 21 patients) versus 52.6% (10 of 19 patients) in the CG (p=0.046).

**Conclusions:**

Se supplementation during RT was effective to improve blood Se status in Se-deficient cervical and uterine cancer patients, and reduces episodes and severity of RT-induced diarrhoea. This effect was most pronounced and significant in patients with large PTV (> 1302 ml).

## Background

The essential trace element selenium (Se) is needed during the biosynthesis of a number of selenocysteine (Sec)-containing selenoproteins. Se supply by the daily nutrition varies considerably between populations, being replete in large areas of the US and rather deficient or sub-optimal in many areas of central Africa, Asia and whole Europe. Among the functionally characterized selenoproteins are a number of enzymes crucially involved in the central antioxidative systems of the human body, namely the glutathione peroxidase (GPx) and the thioredoxin reductase (TRR) isoenzymes. In a number of epidemiologic analyses, Se deficiency has been associated with increased infection risk and adverse mood states**.** Se has been shown to possess cancer-preventive and cytoprotective activities in both animal models and humans. It is well established that Se has a key role in redox regulation and antioxidant function, and hence in membrane integrity, energy metabolism and protection against DNA damage. These and other functions are mediated through a small number of selenoproteins encoded by 25 separate human genes [1-4].

Radio- and chemotherapy, disease-dependent alterations of metabolism as well as the suboptimal nutrition of cancer patients in the clinics might aggravate the situation in a Se-deficient patient even further, and increase the likelihood of clinically relevant Se deficiency and radiation-induced side effects [5-7].

Preliminary clinical evidence indicates that Se functions as a radio- and chemoprotector with the ability to alleviate side effects of tumour specific chemotherapy or radiotherapy treatments [8-11].

In 2003, an English retrospective study indicated a positive correlation between initial serum Se levels and the dose delivery of chemotherapy and outcome in patients with aggressive non-Hodgkin´s lymphoma. In light of the results, the authors hypothesized that specific selenocompounds including methylseleninic acid and selenodiglutathione were involved in inducing preferential cell death in lymphoma cell lines and primary lymphoma cultures, which may be partly attributable to an increased generation of reactive oxygen species [12,13].

The German Working Group Trace Elements and Electrolytes in Oncology (AKTE) conducted the worldwide sole randomized phase III clinical studies to examine the radioprotective properties of sodium selenite in radiation oncology. The first of these randomized trials enrolled head and neck tumour patients undergoing radiation therapy [14]. A total of 39 patients were randomized into two groups. 22 were enrolled in the selenium group (SeG) and 17 in the control group (CG). Weekly analyses were performed and a significant reduction of dysphagia in the SeG at the last week of irradiation (p=0.04) was observed. This small randomized trial had shown some limited effects of Se in the prevention of ageusia (loss of taste) and dysphagia due to radiotherapy because of head and neck cancer. Most likely due to the small number of patients included a clinical relevant radioprotection was not observed [14].

In contrast to these data the second randomized phase III study in postoperative gynecological patients with supplementation of Se under radiotherapy conditions supported the findings of Last et al. [12]; especially the patients with higher plasma and whole blood Se levels tolerated the side effects of radiotherapy significantly better without any obvious impairment of survival data. The main result of this study showed a significant benefit of sodium selenite supplementation with regard to Se deficiency and RT-induced diarrhoea in patients with cervical and uterine cancer [15].

Aim of this study was to perform a subgroup analysis of the data available from the initial study [15]. Specifically, we wanted to examine whether there is a relation between the PTV size and the Se effect concerning radiation induced diarrhoea.

### Patients and methods

The study had been approved by the International Ethics Committee in Freiburg, Germany (No. 2000-D-7251). Each patient had given written informed consent before being accrued. The study had been performed in full accordance with the Declaration of Helsinki.

Patients were also assured of anonymity and of confidentiality of person-related data. Besides, they were assured that refusal to participate in the study would not affect their future care in any way.

### Patients

Between January 2000 and June 2006 whole blood Se concentrations were measured in patients with histopathologically confirmed cervical and endometrial cancer after surgical treatment. Patients with initial Se concentrations of less than 84 μg/l were considered as Se-deficient and randomized into the two study groups.

### Treatment

External RT was delivered with a 6–18 MV linear accelerator. Five fractions per week were planned. Treatment was given with a 3- to 4-field box technique. RT was given as 3D conformal radiotherapy. CT-based treatment planning was performed for all cases. The Clinical Target Volume (CTV) encompassed the primary tumor region and the pelvic regional lymph nodes.

During radiotherapy, patients in the verum group received 500 μg of Se (as inorganic sodium selenite; selenase®) per os on the days of RT and 300 μg of Se on the days without treatment until the last day of radiotherapy. In the control group, adjuvant RT was given without supplementation of Se.

### Measurement of whole blood selenium

During treatment, levels of whole blood Se were measured after completing 50% of RT, and at the end of RT, by means of atomic absorption spectroscopy according to the method of Winnefeld et al. [16].

### Check-ups during treatment

Patients were interviewed and examined by a physician on a weekly basis. Concerning radiation-induced diarrhoea, patients recorded the daily number of bowel movements and scored the stool consistency. With the help of these information diarrhoea was graded weekly according the Common Toxicity Criteria system (CTC, Version 2a).

### Planning Target Volume (PTV)

The Planning Target Volume (PTV) is a geometrical concept, and it is defined to select appropriate beam size and beam arrangements, taking into consideration the net effect off all possible geometrical variations and inaccuracies in order to ensure that the prescribed dose is actually absorbed in the Clinical Target Volume (CTV). The evaluation of the PTV was ascertained with the help of a specialised computer-assisted treatment planning software used for radiation planning procedure.

### Endpoint

The endpoint of this subgroup analysis was to evaluate the frequency of radiation induced diarrhoea depending on Se supplementation and PTV.

### Statistics

All data were stored and analyzed using the SPSS statistical package 19.0 (SPSS Inc., Chicago, Illinois, USA). Descriptive statistics were computed for continuous and categorical variables. The statistics computed included mean and standard deviations of continuous variables and are presented as mean±SD, frequencies and relative frequencies of categorical factors. Testing for differences of continuous variables between the groups was accomplished by the 2-sample *t* test for independent samples or the Mann–Whitney *U* test, as appropriate. For categorical variables comparisons were done by using Fisher´s exact test. The period of time to diarrhoea was estimated using Kaplan-Meier method. Differences between curves were assessed by Mantel´s log-rank test for censored data. All p-values are resulting from two-sided statistical tests and values of p<0.05 were considered to be statistically significant.

## Results

### Patients

A total of 81 patients (age: 64.3 ±10.1, range: 31–80) with carcinomas of cervix (n=11) or corpus uteri (n=70) with a significant whole blood Se deficiency after curative surgical treatment were randomized as described earlier [15]. Accordingly, 39 were enrolled in the SeG and 42 in the CG. Excluded from randomization were patients with metastatic disease, diarrhoea before radiotherapy, radiochemotherapy, supplementation of Se before radiotherapy, and patients who had undergone previous pelvic radiotherapy. Table 1 specifies the main baseline characteristics of the 81 patients concerning the endpoint of the current study. Additional data concerning histology, tumor stage, treatment before radiotherapy and brachytherapy have been published earlier [15].

**Table 1:** Baseline characteristics of the 81 patients enrolled in the subgroup analysis

	**with selenium**	**with selenium**	**p-value**
Number	39(100%)	42(100%)	
Age(Years), Median, Range	64.8(37-80)	63.8(31-80)	0.67
Cervix Uteri	4(10.2%)	7(16.6%)	
Corpus Uteri	35(89.8%)	35(83.4%)	0.52
Total Dose of External			
Radiotherapy in Gy	46.3(39.6-54.0)	48.0(39.6-59.2)	0.06
Planning Theatment Volume	1604(923-4608)	1447(916-3360)	0.55
(PTV)in ml			

### Whole blood Se levels

Before RT, the mean Se level in the SeG was 65.3 ± 13.6 μg/l and in the CG 63.2 ± 12.7 μg/l (p=0.49). Compared to the CG we found a significant increase in the SeG after completing 50% of RT (67.3±16.6 μg/l vs. 93.2±26.0 μg/l, p<0.001). At the end of RT, the mean Se level was 90.9±19.9 μg/l in the SeG and 61.4±15.5 μg/l in the CG (p<0.001) (Table 2).

**Table 2:** Whole blood Se levels in μg/l (means + 95% CI) depending on Se supplementation

	**with selenium**	**with selenium**	**p-value**
	**(Mean+95% of CI)**	**(Mean+95% of CI)**	
Before RT	65.3(60.7-70.0)	62.2(59.4-68.2)	=0.49
50% of RT	93.2(83.8-102.7)	67.3(61.6-72.5)	<0.001
end of RT	90.9(81.3-95.1)	61.4(56.4-66.9)	<0.001

### Incidence of at least CTC 2 radiation induced diarrhoea

The actuarial incidence of radiation-induced diarrhoea of at least CTC 2 in the SeG was 20.5% (8 of 39 patients) compared to 44.5% in the CG (18 of 42 patients) (p=0.04).

### PTV

The median PTV in both groups was 1302 ml (916-4608). The mean PTV in the SeG was 1604 ml compared to 1447 ml in the CG (p=0.55).

### Incidence of at least CTC 2 diarrhoea depending on PTV

**Figure 1:**
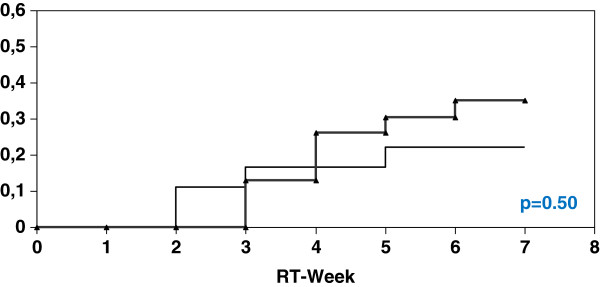
Univariate analysis (log-rank) for the incidence of at least diarrhoea CTC 2 depending on PTV <= 1302 ml (thick curve: without Se; thin curve: with Se).

**Figure 2:**
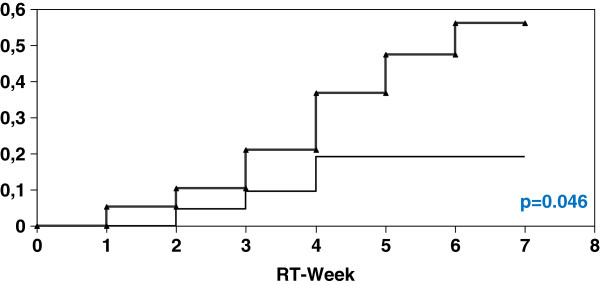
Univariate analysis (log-rank) for the incidence of at least diarrhoea CTC 2 depending on PTV > 1302 ml (thick curve: without Se; thin curve: with Se).

With a PTV of <= 1302 ml (n=41) the actuarial incidence of at least CTC 2 diarrhoea in the SeG was 22.3% (4 of 18 patients) compared to 34.8% in the CG (8 of 23 patients) (p=0.50). In contrast to those patients with a PTV of > 1302 ml (n=40) the actuarial incidence of at least CTC 2 diarrhoea in the SeG was 19.1% (4 of 21 patients) versus 52.6% (10 of 19 patients) in the CG (p=0.046) (Figures 1, 2).

Results on the distribution of the grading scores of diarrhoea according to CTC have been published and are available for comparison [15].

## Discussion

Our study analyzes the effects of Se supplementation in a group of patients with initially low Se status after surgery during RT treatment depending on PTV-size of radiation therapy. Se supplementation improved the Se status of our patients under the therapy conditions, and yielded a significant reduction of diarrhoea as published recently [15]. The increased biosynthesis and hence activities of the different GPx and TRR isoenzymes might have been responsible for these effects by an enhanced neutralization of radiation-induced hydroperoxides and free radicals in the small intestinal mucosa included in the irradiation volume [5,17]. Beside the antioxidative capacity in the healthy tissue a selective unusual activation of wild-type p53 by Se-dependent reduction of two critical cysteine residues by redox factor 1 (Ref 1) might have contributed to the consecutive activation of DNA-repair in healthy cells of the small intestinal mucosa [18-21]. Furthermore, an increased biosynthesis of selenoprotein P, which is responsible for Se homeostasis, transport of selenium to tissues, antioxidant activities and decrease of lipid hydroperoxides, might have further provided some protection to the healthy cells in the irradiated tissue [22]. In addition, Se may have reduced translocation of the inflammatory transcription factor NFkB into the nucleus and thereby diminished cytokine production and release [23-25]. Finally, Se might have stimulated the regulatory mechanisms of repopulation into the small intestinal mucosa included in the irradiation volume at a time before they were activated by irradiation [26].

Here, we provide results indicating that the radioprotective effect of Se concerning radiation induced diarrhoea achieved significance primarily in patients with PTV > 1302 ml. Thus, it can be speculated that in correlation to a growing PTV a threshold concerning the number of radiation induced free radicals exists. As a consequence, it is conceivable that in patients with PTV <=1302 ml the endogenous detoxification system works sufficiently without the help of supplemental Se. In comparison, in cases with PTV >1302 ml and consecutively expected higher count of radiation induced free radicals we observed that Se supplementation yielded a significant prevention of diarrhoea. This finding may be due to a higher demand for Se-dependent protective enzymes and an exhaustion of the Se reserve in the tumour patients if not efficiently supplemented during RT.

Unfortunately, in 2011 there is still no general recommendation in favour of or against Se supplementation for alleviating the side effects of chemotherapy, radiotherapy and surgery in cancer patients [27]. Nevertheless, our results support the published findings of Last et al. [12] and highlight that in patients with higher blood selenium levels the tolerance of radiotherapy was significantly better. Therefore, we strongly advocate to take the Se status of the tumour patients under therapy more seriously into account and to consider a respective supplementation prior to therapy when the current Se status appears to be insufficient [28,29].

## Conclusions

Se supplementation during RT of the pelvic region is effective to improve blood Se status in Se-deficient cervical and uterine cancer patients, and reduces episodes and severity of RT-induced diarrhoea. This effect was most pronounced and significant in patients with large PTV (> 1302 ml).

## Abbreviations

CG-Control: Group; CTC-Common: Toxicity Criteria System; GPx-Glutathion: Peroxidase; PTV-Planning: Target Volume; RT-Radiotherapy: Se-Selenium; Sec-: Selenocysteine; SeG-Selenium: Group; TRR-Thioredoxin: Reductase

## Competing interests

RM has had conference and travel expenses reimbursed and has received lecture fees from the pharmaceutical company biosyn-Arzneimittel GmbH, biosyn-Arzneimittel GmbH is not financing this manuscript. The other authors declare that they have no competing interests.

## Authors’ contributions

RM, OM, JB, MG, DB and RBS have made substantial contributions to conception and design of the study. RM, MG, DB, RBS, FJP and BR have made substantial contributions to acquisition of data. RM, OM, LS, GK and JB have made substantial contributions to analysis and interpretation of data. RM and OM have been involved in drafting the manuscript. LS, JB, KK, US, JH, HE and IAA revised it critically for important intellectual content. All authors have given final approval of the version to be published.
